# A Case of One-Stage Surgical Treatment for a Bladder Hernia with Bladder Calculi

**DOI:** 10.70352/scrj.cr.25-0558

**Published:** 2026-01-20

**Authors:** Naoki Kubo, Norihiko Furusawa, Harunari Fukai, Masaru Terada

**Affiliations:** Department of Surgery, Nagano Prefectural Shinshu Medical Center, Suzaka, Nagano, Japan

**Keywords:** bladder hernia, bladder calculi, inguinal hernia

## Abstract

**INTRODUCTION:**

A bladder hernia associated with bladder calculi is an extremely rare condition, with no consensus established regarding its management. Herein, we report a case in which 1-stage surgical treatment was performed for a bladder hernia complicated by bladder calculi.

**CASE PRESENTATION:**

The patient presented with a swelling in the right inguinal region. CT revealed a bladder hernia accompanied by multiple bladder calculi. An inguinal incision was made, the bladder stones were removed, and hernia repair was performed using the Lichtenstein method. At 3 years postoperatively, there was no recurrence of either bladder calculi or hernia.

**CONCLUSIONS:**

For inguinal hernias accompanied by bladder calculi in which transurethral lithotripsy is challenging, open surgical fragmentation of bladder calculi and inguinal hernia repair by using the Lichtenstein method through the same surgical field may be useful approaches.

## INTRODUCTION

Bladder hernias accompanied by bladder calculi are extremely rare, with only 11 cases, including the present case, reported to date.^1–9)^ Although endoscopic lithotripsy has recently become the standard treatment for bladder calculi,^10)^ in cases complicated by an inguinal hernia, as in the present case, open lithotomy allows for simultaneous hernia repair through the same surgical field. Herein, we report the case of a bladder hernia with multiple bladder calculi, in which the herniated bladder was incised, lithotomy was performed, and 1-stage hernia repair was successfully achieved.

## CASE PRESENTATION

**Fig. 1:**
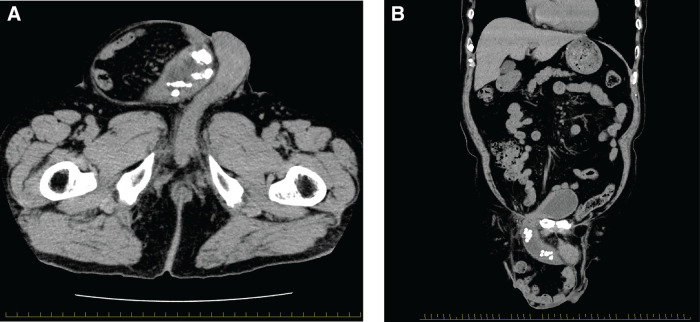
(**A**) CT image of a bladder stone in the herniated bladder, coronal section. (**B**) CT image of a bladder stone in herniated bladder, axial section.

An 84-year-old male patient presented with a 10-year history of right inguinal swelling and urinary frequency. His past medical history included hypertension and appendectomy. Physical examination revealed a large right scrotal inguinal hernia and a high urinary frequency. Digital rectal examination revealed an enlarged prostate with a smooth surface. CT demonstrated herniation of the bladder and small intestine into the right scrotal sac, with multiple bladder calculi, which measured approximately 10 mm within the herniated bladder (**Fig. 1A** and **1B**). Laboratory investigations, including blood tests and urinalysis, revealed no evidence of infection. Given that the urological assessment indicated that endoscopic lithotripsy would be difficult, a surgical plan was made to approach the bladder stones and repair the hernia via an inguinal incision. An incision was made from the right inguinal region to the scrotum, and a direct inguinal hernia was detected. The hernia defect measured 4 cm in diameter and was classified as M3 based on the European Hernia Society (EHS) classification.

**Fig. 2:**
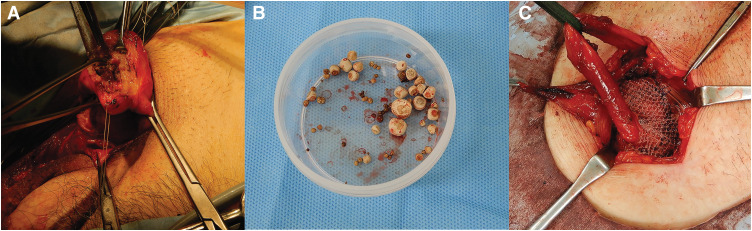
(**A**) The bladder was incised, and the stones were removed. (**B**) The stones removed from the bladder. (**C**) Hernia repaired by using the Lichtenstein method.

The herniated bladder body was incised, and all of the bladder calculi were removed (**Fig. 2A** and **2B**). The bladder was repaired in a single step and anatomically repositioned. Specifically, the bladder was repaired with a 2-layer closure. Additionally, the hernia defect in the transversalis fascia was closed with continuous suture.

After changing gloves and thoroughly irrigating the surgical field, the inguinal hernia was repaired by using the Lichtenstein technique (**Fig. 2C**). During the 3-year postoperative follow-up, no recurrence of either bladder calculi or hernia was observed.

## DISCUSSION

In the present case, we performed open lithotripsy and hernia repair (Lichtenstein method) through the same surgical field for a bladder hernia accompanied by bladder calculi. Bladder hernias associated with bladder calculi are extremely rare. A search of PubMed with the keywords “bladder hernia” and “bladder stones or calculi” revealed only 11 cases, including the present case.^1–9)^

Bladder hernias account for approximately 1%–5% of all inguinal hernias, and typically occur on the right side. The reported risk factors include advanced age, male sex, obesity, weakness of the abdominopelvic musculature, and conditions that increase intravesical pressure, such as benign prostatic hyperplasia (BPH) and neurogenic bladder.^11,12)^ All reported cases of inguinal bladder hernias with bladder calculi occurred in male patients, with 10 of the 11 cases involving the right side. In all the patients, symptoms such as voiding dysfunction, 2-stage voiding, and lower urinary tract obstruction were observed. The bladder had herniated into the scrotum, and it is believed that significant urinary stasis contributed to the formation of bladder calculi.

Although transurethral endoscopic lithotripsy is generally the standard treatment for bladder calculi, open surgery is considered when large or multiple stones are present, or when concomitant surgical procedures for conditions such as an inguinal hernia or tumors are needed.^10)^ Among the 9 reported cases in which lithotripsy was used, 5 underwent open lithotomy.

The advantage of open lithotomy in these cases is the ability to perform lithotomy and hernia repair simultaneously through the same surgical field. However, such cases should be managed collaboratively by urologists and general surgeons. A 2-stage approach, in which endoscopic lithotripsy would be performed after hernia repair, was also considered. However, in this case, multiple bladder calculi and significant prostatic enlargement were present, and the urologists determined that transurethral lithotripsy after bladder reduction and hernia repair would be technically difficult. Therefore, open lithotripsy was selected.

In 7 out of 8 cases in which hernia repair was described mesh was used, and in 6 out of 7 cases in which the surgical technique was specified, including the present case, the repair was performed using the Lichtenstein method.

This method may reduce the risk of infection and recurrence of bladder calculi compared with other approaches, including anterior preperitoneal and laparoscopic repairs, by minimizing direct contact between the bladder and the mesh.^13,14)^ However, due to the fact that the procedure involves operating in the same surgical field as the bladder stone removal, there is a potential risk of infection. In the present case, due to the fact that no signs of infection were observed on preoperative blood tests or urinalysis, we selected the Lichtenstein method to perform hernia repair in a single stage through the same surgical field.

In our case, no recurrence of bladder calculi or hernia, nor any surgical site infection, was observed. However, in cases with preoperative urinary tract infection, hernia repair through the same surgical field may demonstrate a higher risk of infection, and laparoscopic repair or a 2-stage procedure should be considered.

Moreover, even in cases in which lithotripsy was performed transurethrally, the Lichtenstein method was selected for hernia repair, possibly to avoid direct mesh-to-bladder contact as much as possible.

Preoperative CT evaluation is recommended for bladder hernias to minimize the risk of bladder injury during surgery.^15,16)^ However, the optimal surgical approach—whether anterior or laparoscopic—remains controversial. With the increasing adoption of laparoscopic techniques for inguinal hernia repair, laparoscopic management of bladder hernias is becoming more common, as it offers the potential advantage of reduced bladder injury. Nevertheless, comparative outcomes between laparoscopic and anterior approaches remain unclear.^17,18)^ In the future, if transurethral lithotripsy is feasible, laparoscopic hernia repair may be a viable option. Further accumulation of cases is needed to better define the optimal management strategy.

Although the use of the Lichtenstein method in our case was effective, laparoscopic hernia repair may represent a promising option in the future. However, further accumulation of cases is required to determine the optimal treatment strategy according to the approach for bladder stone removal and the preoperative condition of the urinary tract.

## CONCLUSIONS

For inguinal hernias accompanied by bladder calculi in which transurethral lithotripsy is challenging, open surgical fragmentation of bladder calculi and inguinal hernia repair by using the Lichenstein method through the same surgical field may be useful approaches.

## DECLARATIONS

### Availability of data and materials

Data sharing is not applicable to this article.
